# Pauson-Khand Reaction of Internal Dissymmetric Trifluoromethyl Alkynes. Influence of the Alkene on the Regioselectivity

**DOI:** 10.3390/molecules19021763

**Published:** 2014-02-03

**Authors:** Nuria Aiguabella, Elsa M. Arce, Carlos del Pozo, Xavier Verdaguer, Antoni Riera

**Affiliations:** 1 Institute for Research in Biomedicine (IRB Barcelona), Baldiri i Reixac, 10. 08028 Barcelona, Spain; E-Mails: nuria.aiguabella@irbbarcelona.org (N.A.); elsa_mtz@hotmail.es (E.M.A.); xavier.verdaguer@irbbarcelona.org (X.V.); 2 Department de Química Orgánica, Universidad de Valencia, E-46100 Burjassot, Spain; E-Mail: Carlos.Pozo@uv.es; 3 Laboratorio de Moléculas Orgánicas, Centro de Investigación Príncipe Felipe, E-46012 Valencia, Spain; 4 Department de Química Orgànica, Universitat de Barcelona, Martí i Franqués, 1. 08028 Barcelona, Spain

**Keywords:** Pauson-Khand reaction, cycloadditions, regioselectivity, cyclopentenones, trifluoromethylalkynes

## Abstract

The scope of the Pauson-Khand reaction (PKR) of internal trifluoromethyl alkynes, previously described with norbornadiene, is expanded to norbornene and ethylene. A thorough structural analysis of the resulting PK adducts has been carried out to unveil that α-trifluoromethylcyclopentenones are preferred in all cases, independently of the electronic properties of the alkyne. The regioselectivity observed with norbornadiene and ethylene is higher than in the case of norbornene.

## 1. Introduction

The Pauson-Khand reaction (PKR) is a cobalt catalyzed [2+2+1] cycloaddition between an alkene, an alkyne and carbon monoxide to give a cyclopentenone [1]. The intramolecular PKR has been extensively used in organic synthesis since both the regio- and the stereochemical outcome of the reaction are substrate-controlled. However, the intermolecular PKR has been less exploited mainly because of the small range of reactive alkenes. The intermolecular reaction with terminal alkynes is completely regioselective affording adducts with the substituent always in α-position of the enone. In the case of internal dissymmetric alkynes, the regioselectivity of the PK adducts depends on a combination of steric and electronic effects [2,3,4,5,6,7]. In the absence of steric effects, electron-donating groups (EDGs) show preference for the α-position whereas electron-withdrawing groups (EWGs) tend to place at the β-position [3,4,5]. However, recent studies have shown that the electronic effects are less significant than previously described [3,4,5] and can, therefore, be overcome by steric effects [6,7]. In this context, we became interested in exploring the reactivity of dissymmetric fluorinated alkynes in the intermolecular PKR in order to uncover how the particular electronic and steric properties of these substrates affect the regiochemical outcome of the intermolecular PKR [8,9].

The remarkable physicochemical properties [10] of fluorinated compounds has led to an increasing effort to the development of reliable methods for their preparation and to an exploration of the effect that fluorinated substituents have on reactivity [11,12]. In spite of the great development that fluorine chemistry has experienced during the last decade, only a few examples of the PKR of fluorinated alkynes have been reported to date. The first examples of fluorinated alkynes in the intramolecular PKR were reported at the beginning of the past decade [13,14,15,16,17,18]. In 2010 the first example of intermolecular PKR of fluorinated alkynes with norbornadiene was reported [8]. The most striking result from this study was the fact that the PKR of fluorinated alkynes was extremely regioselective leading, unexpectedly, to the α-fluorinated cyclopentenones. These observations were further confirmed recently, when we described [9] the PKR of a large set of internal trifluoromethyl alkynes bearing electron-donating and electron-withdrawing groups with norbornadiene. With no exception, only the α-trifluoromethylcyclopentenone was obtained in good yield. Given that the electronic effect should direct the most electron-withdrawing substituent to the β-position, we hypothesized that it was being overridden by the steric hindrance of the trifluoromethyl group. On the other hand, Konno and co-workers [19] described the PKR of several trifluoromethyl alkynes with norbornene instead of norbornadiene and reported that the regioselectivity of the PKR was not complete. To confirm these findings and to shed some light into this somewhat surprising difference between norbornene and norbornadiene, we sought to study the PKR of internal dissymmetric alkynes with norbornene and ethylene in order to understand how the alkene can influence the regioselectivity of the PKR.

## 2. Results and Discussion

**Figure 1:**
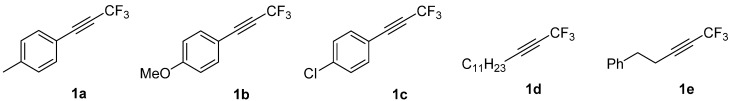
Dissymmetric fluorinated alkynes used in this study.

In order to cover a wide range of electronic properties, we chose trifluoromethyl alkynes **1a**–**d** containing electron-donating and electron-withdrawing substituted aromatic rings and aliphatic chains (Figure 1). The cobalt hexacarbonyl complexes **2a**–**d** of these alkynes were synthesized from the corresponding terminal alkynes following the methodology described by us [9] based on Qing’s trifluoromethylation reaction [20,21].

**Figure 2:**
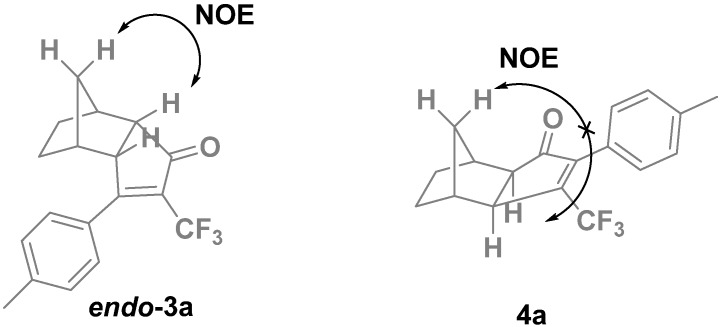
Characteristic NOE's for the *endo* diastereomers that are not present in the *exo* isomers.

We first carried out the PKR between the cobalt complex of *p*-tolyltrifluoromethylacetylene (**2a**) and norbornene under typical thermal conditions. The reaction proceeded smoothly and gave the PK adducts in an excellent 87% yield as an 86:14 mixture of isomers (Table 1, entry 1). The structure of the major isomer **3a** was easily determined by NMR and confirmed by chemical correlation: the same compound was obtained from the PK adduct of **2a** with norbornadiene by catalytic hydrogenation (Pd/C, H_2_, MeOH). The structure of the minor isomer was more difficult to ascertain, since it could be either the regioisomer **4a** or the *endo* isomer *endo*-**3a** (Figure 2). Careful study by NMR allowed us to rule out the *endo* isomer. One diagnostic observation was the absence of the NOE correlations expected for *endo*-**3a** (Figure 2). Therefore, we could confirm that the minor PK adduct was the regioisomer **4a** with the CF_3_ at the β-position. It is worth to mention that the PKR of the same substrate with norbornadiene is completely regioselective.

The alkyne cobalt complexes **2b**-**c** were submitted to the same reaction conditions and both ^1^H and ^19^F-NMR of the crude reaction products also showed two sets of signals. Careful purification and analysis of their NMR spectra showed that the major isomers were the α-fluorinated compounds **3b**–**c** whereas the minor isomers were the β-fluorinated PK adducts (Table 1). However, in the case of **3d** the presence of the minor regioisomer was marginal, so it could only be detected by ^19^F-NMR. These results are in agreement with those reported by Konno and co-workers [19]. The regioselectivities we detect for the PKR of **2b** and **2c** are in concordance with their results for the same substrates. The spectroscopical data is also coincident with the one given in their report, although in our case we provide complete characterization for all the major and minor diastereomers for the first time.

The fact that the regioselectivity was poorer with an electron-withdrawing substituent in the alkyne (compound **2c**) than with electron-donating substituents (compounds **2a**–**b**) adds more difficulty in understanding the regiochemistry of the PKR. If we assume that the trifluoromethyl group will preferentially occupy the α-position in the final product mainly for steric reasons, we could argue that this tendency should be reinforced when having an electron-withdrawing substituent partner, since it would prefer the β-position, and diminished with an electron-donating group, that would compete for the α-position. Theoretical studies on the regiochemistry of these reactions are ongoing in our laboratory in order to explain these results.

**Table 1:** Regioselectivity of the PKR of trifluoromethyl alkynes **1a**–**d** with norbornene. 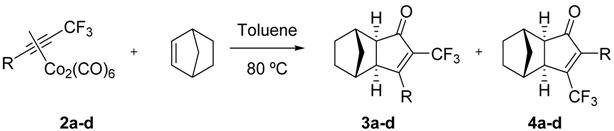

Entry	SM	R	Product	Yield (%)	Ratio 3:4
1	**2a**	*p*-MePh	**3a, 4a**	87	86:14
2	**2b**	*p*-MeOPh	**3b, 4b**	51	86:14
3	**2c**	*p*-ClPh	**3c, 4c**	41	71:29
4	**2d**	C_11_H_23_	**3d**	40	>95:5

Once explored the reactivity of norbornene in the PKR, we tackled the PKR of dissymmetric fluorinated alkynes with ethylene [2], tetramethylnorbornadiene [22], and (*E*)-cyclooctene [23], all of them used as alkenes in the PKR. As for the PKR with norbornene and in order to cover a representative range of electronic properties, we selected the same alkynes **1a**–**c** as before. However, instead of the alkyne 1d with an aliphatic side chain, we selected ethylphenyltrifluoromethylacetylene (**1e**) in order to decrease the volatility and lipophilicity of the final product and therefore simplify its isolation and handling.

We first carried out an optimization of the ethylene reaction with the cobalt complex **1a** as a model. The beneficial effect of the use of molecular sieves as an additive for the intermolecular PKR is well-established [24,25], and our group had already experienced how the use of this zeolite in PKRs with ethylene increased the reaction yields dramatically [2]. At sight of these precedents, we decided to include molecular sieves in our optimization reactions. These zeolites are supposed to promote the PKR by increasing the availability of ethylene in the solvent and by acting as a micro-reactor. In order to further increase the presence of ethylene in the liquid sine of the reaction, we decided to carry out the reaction at low temperature and to activate it with *N-*methylmorpholine-*N*-oxide (NMO), the addition of which was done slowly in order to prevent decomposition of the starting cobalt-alkyne complex (Table 2).

The yields obtained were very low in all cases when activating the reaction by the addition of NMO. The use of molecular sieves did not improve the reaction yield (entry 1), and our attempts to carry out the reaction at lower temperature to slow down the oxidation of our cobalt complex and to increase the solubility of ethylene were fruitless (entries 2, 3).

**Table 2:** PKR of trifluoromethyl alkynes **1a**–**e** with ethylene. 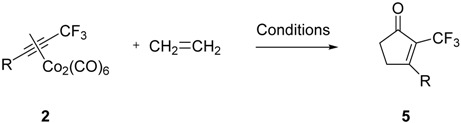

Entry	SM	R	Conditions	Product	Yield
1	**1a**	*p*-MePh	6 bars ethylene, NMO ^b^ (10 equiv), 4 Å MS (activated ^a^; 15x alkyne mass), rt, DCM	**5a**	18%
2	**1a**	*p*-MePh	6 bars ethylene, NMO ^b^ (10 equiv), rt, DCM	**5a**	25%
3	**1a**	*p*-MePh	6 bars ethylene, NMO ^b^ (10 equiv), 0 °C, DCM	**5a**	9%
4	**1a**	*p*-MePh	6 bars ethylene, 70 °C to 80 °C, toluene	**5a**	53%
5	**1b**	*p*-MeOPh	6 bars ethylene, 80 °C to 85 °C, toluene	**5b**	58%
6	**1c**	*p*-ClPh	6 bars ethylene, 80 °C to 85 °C, toluene	**5c**	56%
7	**1e**	PhC_2_H_4_	6 bars ethylene, 80 °C to 85 °C, toluene	**5e**	23%

^a^ dried at 200 °C under high vacuum for 8 h, then stored in a vacuum oven; ^b^ slow addition; rate: 1 equiv./20 min.

Our best result was obtained when carrying out the reaction thermally and without the presence of molecular sieves (entry 4). Once explored the reaction conditions, we proceeded to test the remaining selected substrates (entries 5–7). Even though the yields were moderate, the crude reaction mixtures were very clean and the final products very easy to purify. In all cases only one PK adduct could be recovered upon purification and, after careful NMR analysis, all of them were assigned as the α-trifluoromethylcyclopentenones. Unfortunately, the reactions with other olefins were unsuccessful; complex **2d** showed no reactivity with tetramethylnorbornadiene [22] after 24 h at 80 °C, whereas complex **2c** decomposed in the presence of (*E*)-cyclooctene [23] and NMO in methylene chloride.

Removal of the trifluoromethyl group from the norbornadiene PK adducts allowed us the preparation of the previously unknown regioisomeric PK adducts of terminal alkynes. [8] Therefore, the trifluoromethyl group was used as a steering group to control the regioselectivity of the PK. With the new norbornene PK adducts in hand we wanted to test the generality of the process. Following the conditions previously established by our group [8,9], the reactions proceeded smoothly and, even though the yields were only moderate, they were clean in general and the products could be easily purified. The only exception was adduct **3d** since, even though the final product could be detected by NMR and isolated, it was accompanied by unidentified reaction by-products. The minor regioisomers were also submitted to these reaction conditions and no evolution could be observed in any case, which represents a further confirmation that the minor adducts correspond, as expected, to the β-trifluoromethyl regioisomers and not to the diastereomeric *endo* α-trifluoromethyl products. PK adducts with ethylene **5a**–**d** were also submitted to the destrifluoromethylation reaction conditions (Table 3). However, probably due to the volatility of the final products, they could not be isolated.

**Table 3:** Removal of the trifluoromethyl group form the norbornene PK adducts. 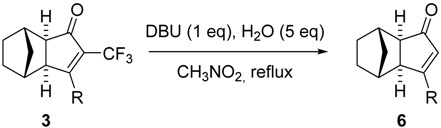

Entry	SM	R	Product	Yield (%)
1	**3a**	*p*-MePh	**6a**	28
2	**3b**	*p*-MeOPh	**6b**	49
3	**3c**	*p*-ClPh	**6c**	30
4	**3d**	C_11_H_23_	**6d**	nd

## 3. Experimental

### 3.1. General

Non-aqueous reactions were carried out under nitrogen atmospheres and with flame-dried glassware. Dry toluene was purchased directly from Sigma-Aldrich (St. Louis, MO, USA). Commercially available reagents were used with no further purification. All reactions were monitored by TLC analysis using Merck 60 F_254_ silica gel on aluminum sheets. Silica gel chromatography was performed by using 35-70 µm silica or an automated chromatography system (Combiflash®, Teledyne Isco, Lincoln, NE, USA) with hexanes/ethyl acetate gradients as eluent, unless noted otherwise.

NMR spectra were recorded at room temperature on a Varian Mercury 400 or a Bruker 300 instrument. ^1^H and ^13^C-NMR spectra were referenced to the residual peaks of the deuterated solvent. ^19^F-NMR spectra were referenced by the spectrometer without any external pattern. The following abbreviations were used to define the multiplicities: s, singlet; d, doublet; t, triplet; q, quadruplet; p, quintuplet; m, multiplet; br s, broad signal. The chemical shifts (*δ*) are expressed in ppm and the coupling constants (*J*), in hertz (Hz). IR spectra were recorded in a Thermo Nicolet Nexus FT-IR apparatus, either by preparing a KBr disk or by depositing a film of the product on a NaCl window. Absorptions are given in wavenumbers (cm^−1^). Melting points were recorded in a Büchi B-540 apparatus. Mass spectrometry analysis was performed as high resolution ESI analysis.

### 3.2. General Conditions for the Pauson-Khand Reaction with Norbornene

The cobalt complexes **2** were prepared according to reference 9. The cobalt complex was dissolved in anhydrous toluene (4 mL/0.1 mmol complex) in a round bottom flask equipped with a magnetic stirrer and put under N_2_. Norbornene (10 eq.) was then added, and the system was heated up to 80 °C. The reaction was allowed to progress for 24 h. After that time, the solvent was removed under reduced pressure. The crude was purified by silica gel chromatography using mixtures of hexanes/AcOEt of increasing polarity.

(3aS*,4R*,7S*,7aR*)-3-(4-Tolyl)-2-(trifluoromethyl)-3a,4,5,6,7,7a-hexahydro-1H-4,7-methanoinden-1-one: The general procedure was followed starting from cobalt complex **2a** (75 mg, 0.15 mmol) and heated at 80 °C for 24 h. The desired products were isolated as white solids (36 mg, 87%) and as a 5.9:1 mixture of regioisomers that could be separated by silica gel chromatography. Major regioisomer **3a**: Mp: 94–96 °C. IR (KBr) *ν*_max._ = 2961, 2874, 1705, 1618, 1364, 1124, 983, 831 cm^−1^. ^1^H-NMR (400 MHz, CDCl_3_) *δ* = 1.01 (dp, *J* = 10.8 and 1.4 Hz, 1H), 1.12 (dp, *J* = 10.7 and 1.9 Hz, 1H), 1.35 (m, 2H), 1.63 (m, 2H), 2.00 (dd, *J* = 3.0 and 1.5 Hz, 1H), 2.42 (s, 2H), 2.45 (dt, *J =* 5.6 and 1.1 Hz, 1H), 2.60–2.56 (m, 1H), 3.09 (ddd, *J* = 4.9, 3.0 and 1.6 Hz, 1H), 7.31–7.24 (m, 4H). ^13^C-NMR (100 MHz, CDCl_3_) *δ* = 21.4 (CH_3_), 28.4 (CH_2_), 28.9 (CH_2_), 31.5 (CH_2_), 38.4 (CH), 39.6 (CH), 52.9 (CH), 54.3 (q, *^4^J_CF_* = 1 Hz, CH), 121.5 (q, *^1^J_CF_* = 273 Hz, C), 127.5 (q, *^5^J_CF_* = 2 Hz, CH), 129.2 (CH), 130.9 (C), 132.0 (q, *^2^J_CF_* = 31 Hz, C), 141.1 (C), 179.1 (q, *^4^J_CF_* = 3 Hz, C), 203.6 (m, C). ^19^F-NMR (376 MHz, CDCl_3_) *δ* = −59.88 (d, *J* = 2.4 Hz, 3F). HRMS (ESI) calculated for C_18_H_18_F_3_O 307.1304, found 323.1303 [M+H]^+^. Minor regioisomer **4a**: Mp: 94–96 °C. IR (KBr) *ν*_max._ = 2958, 1719, 1608, 1512, 1173, 1127 cm^−1^. ^1^H-NMR (400 MHz, CDCl_3_) *δ* = 1.23–1.08 (m, 2H), 1.39 (m, 2H), 1.70–1.59 (m, 1H), 1.84–1.72 (m, 1H), 2.40–2.35 (m, 3H), 2.45 (d, *J* = 5.5 Hz, 1H), 2.56–2.54 (m, 2H), 2.93 (d, *J* = 5.5 Hz, 1H), 7.15 (d, *J* = 8.1 Hz, 2H), 7.21 (d, *J* = 8.2 Hz, 2H). ^13^C-NMR (100 MHz, CDCl_3_) *δ* = 21.4 (CH_3_), 28.3 (CH_2_), 29.0 (CH_2_), 31.3 (CH_2_), 38.2 (CH), 40.1 (CH), 47.2 (q, *^3^J_CF_* = 2 Hz, CH), 53.6 (CH), 124.2 (q, *^1^J_CF_* = 274 Hz, C), 126.0 (C), 128.7 (q, *^5^J_CF_* = 2 Hz, CH), 128.8 (CH), 139.2 (C), 155.4 (C), 207.9 (C). ^19^F-NMR (376 MHz, CDCl_3_) *δ* = −60.08 (s, 3F). HRMS (ESI) calculated for C_18_H_18_F_3_O 323.1304, found 323.1306 [M+H]^+^.

(3aS*,4R*,7S*,7aR*)-3-(4-Methoxyphenyl)-2-(trifluoromethyl)-3a,4,5,6,7,7a-hexahydro-1H-4,7-methanoinden-1-one: The general procedure was followed starting from cobalt complex **2b** (123 mg, 0.25 mmol) and heated at 80 °C for 96 h. The desired products were isolated as colorless oils (35 mg, 51%) and as a 6.4:1 mixture of regioisomers that could be separated by silica gel chromatography. Major regioisomer **3b**: IR (film) *ν_max._* = 2960, 2876, 1713, 1604, 1512, 1175, 839 cm^−1^. ^1^H-NMR (400 MHz, CDCl_3_) *δ* = 1.00 (dp, *J =* 10.8 and 1.4 Hz, 1H), 1.09 (m, 1H), 1.34–1.37 (m, 2H), 1.62–1.64 (m, 2H), 2.00 (s, 1H), 2.45 (dt, *J =* 5.7 and 1.1 Hz, 1H), 2.57 (dd, *J =* 2.6 and 1.4 Hz,1H), 3.12 (ddt, *J =* 5.9, 2.3 and 1.2 Hz, 1H), 3.87 (s, 3H), 6.93–7.02 (m, 2H), 7.37–7.42 (m, 2H). ^13^C-NMR (100 MHz, CDCl_3_) *δ* = 28.5 (CH_2_), 28.9 (CH_2_), 31.6 (CH_2_), 36.7 (CH), 39.6 (CH), 52.5 (CH), 54.2 (q, *^4^J_CF_* = 1 Hz, CH), 55.4 (CH_3_), 114.0 (CH), 121.7 (q, *^1^J_CF_* = 273 Hz, C), 125.7 (C), 129.8 (q, *^5^J_CF_* = 2 Hz, CH), 130.9 (q, *^2^J_CF_* = 25 Hz, C), 161.8 (C), 178.3 (q, *^3^J_CF_* = 3 Hz, C), 203.6 (C). ^19^F-NMR (376 MHz, CDCl_3_) *δ* = −59.77 (d, *J =* 2.4 Hz, 3F). HRMS (ESI) calculated for C_18_H_18_F_3_O_2_ 323.1253, found 323.1251 [M+H]^+^. Minor regioisomer **4b**: IR (film) *ν*_max._ = 2958, 1719, 1608, 1512, 1173 cm^−1^. ^1^H-NMR (400 MHz, CDCl_3_) *δ* = 1.21–1.07 (m, 2H), 1.46–1.31 (m, 2H), 1.64 (tt, *J* = 12.6 and 4.2 Hz, 1H), 1.84–1.71 (m, 1H), 2.44 (d, *J* = 5.5 Hz, 1H), 2.57–2,51 (m, 2H), 2.92 (d, *J* = 5.5 Hz, 1H), 3.82 (d, *J* = 2.9 Hz, 4H), 6.97–6.89 (m, 2H), 7.27–7.19 (m, 2H). ^13^C-NMR (100 MHz, CDCl_3_) *δ* = 28.3 (CH_2_), 29.0 (CH_2_), 31.3 (CH_2_), 38.2 (CH), 40.1 (CH), 47.1 (q, *^3^J_CF_* = 2 Hz, CH), 53.5 (CH), 55.2 (CH_3_), 113.6 (CH), 113.7 (q, *^2^J_CF_* = 14 Hz, C), 128.3 (C), 130.3 (q, *^5^J_CF_* = 2 Hz, CH), 156.1 (q, *^1^J_CF_* = 220 Hz, C), 160.3 (C), 208.1 (C). ^19^F-NMR (376 MHz, CDCl_3_) *δ* = −60.90 (s, 3F). HRMS (ESI) calculated for C_18_H_18_F_3_O_2_ 323.1253, found 323.1255 [M+H]^+^.

(3aS*,4R*,7S*,7aR*)-3-(4-Chlorophenyl)-2-(trifluoromethyl)-3a,4,5,6,7,7a-hexahydro-1H-4,7-methanoinden-1-one: The general procedure was followed starting from cobalt complex **2c** (100 mg, 0.20 mmol) and heated at 80 °C for 72 h. The desired products were isolated as colorless oils (19 mg, 41%) and as a 2.5:1 mixture of regioisomers that could be separated by silica gel chromatography. Major regioisomer **3c**: IR (film) *ν*_max._ = 2961, 1718, 1365, 1188, 1128 cm^−1^. ^1^H-NMR (400 MHz, CDCl_3_) *δ* = 1.01–1.10 (m,1H), 1.17–1.08 (m, 1H), 1.41–1.29 (m, 2H), 1.69–1.60 (m, 2H), 2.04–1.98 (m, 1H), 2.48 (dd, *J* = 5.6 and 1.3 Hz, 1H), 2.63–2.57 (m, 1H), 3.09–3.02 (m, 1H), 7.34–7.27 (m, 2H), 7.49–7.41 (m, 1H) . ^13^C-NMR (100 MHz, CDCl_3_) *δ* = 28.4 (CH_2_), 28.9 (CH_2_), 31.5 (CH_2_), 38.1 (CH), 40.0 (CH), 53.0 (CH), 54.3 (CH), 121.2 (q, *^1^J_CF_* = 274 Hz, C), 128.7 (q, *^5^J_CF_* = 2 Hz, CH), 128.9 (CH), 132.2 (C), 133.0 (q, *^2^J_CF_* = 31 Hz, C), 136.7 (C), 177.2 (q, *^4^J_CF_* = 3 Hz, C), 203.1 (C). ^19^F-NMR (376 MHz, CDCl_3_) *δ* = −59.97 (d, *J* = 2.1 Hz, 3F). HRMS (ESI) calculated for C_17_H_15_ClF_3_O 327.0758, found 327.0759 [M+H]^+^. Minor regioisomer **4c**: IR (film) *ν*_max._ = 2960, 1720, 1492, 1175 cm^−1^. ^1^H-NMR (400 MHz, CDCl_3_) *δ* = 1.12–1.18 (m, 2H), 1.47–1.34 (m, 2H), 1.69–1.60 (m, 1H), 1.84–1.73 m, 1H), 2.47 (d, *J* = 5.5 Hz, 1H), 2.56 (s, 2H), 2.98–2.91 (m, 1H), 7.21–7.17 (m, 2H), 7.40–7.36 (m, 2H). ^13^C-NMR (100 MHz, CDCl_3_) *δ* = 28.2 (CH_2_), 29.1 (CH_2_), 31.3 (CH_2_), 38.2 (CH), 40.2 (CH), 47.3 (q, *^3^J_CF_* = 2 Hz, CH), 53.6 (CH), 124.0 (C), 127.4 (C), 128.5 (C), 130.2 (q, *^5^J_CF_* = 2 Hz, CH), 135.4 (C), 207.3 (C) .^19^F-NMR (376 MHz, CDCl_3_) *δ* = −60.16 (s, 3F). HRMS (ESI) calculated for C_17_H_15_ClF_3_O 327.0758, found 327.0760 [M+H]^+^.

(3aS*,4R*,7S*,7aR*)-2-(Trifluoromethyl)-3-undecyl-3a,4,5,6,7,7a-hexahydro-1H-4,7-methanoinden-1-one (**3d**)*:* The general procedure was followed starting from cobalt complex **2d** (150 mg, 0.28 mmol) and heated at 80 °C for 19 h. The desired product was isolated as a colorless oil (41 mg, 40%). IR (film) *ν*_max._ = 2927, 2855, 1718, 1638, 1367, 1130 cm^−1^. ^1^H-NMR (400 MHz, CDCl_3_) *δ* = 0.91–0.86 (m, 3H), 1.06–1.03 (m, 2H), 1.65–1.60 (m, 20H), 1.78–1.70 (m, 1H), 2.28 (d*, J* = 5.5 Hz, 1H), 2.37 (d*, J* = 4.4 Hz, 1H), 2.43 (m, 1H), 2.50 (d*, J* = 4.1 Hz, 1H), 2.70 (dd, *J* = 5.6 and 2.4 Hz, 1H), 2.85–2.75 (m, 1H). ^13^C-NMR (100 MHz, CDCl_3_) *δ* = 14.1 (CH_3_), 22.7 (CH_2_), 28.3 (CH_2_), 28.51–28.3 (q, *^6^J_CF_* = 1 Hz, CH_2_), 29.2 (CH_2_), 29.3 (CH_2_), 29.3 (CH_2_), 29.4 (CH_2_), 29.6 (CH_2_), 29.9 (CH_2_), 30.5 (q, *^5^J_CF_* = 2 Hz, CH_2_), 31.5 (CH_2_), 31.9 (CH_2_), 38.0 (CH), 39.4 (CH), 51.6 (CH), 54.0 (CH), 122.0 (d, *^1^J_CF_* = 273 Hz, C), 132.7 (q, *^2^J_CF_* = 31 Hz, C), 184.6 (q, *^3^J_CF_* = 3 Hz, C), 203.9 (C). ^19^F-NMR (376 MHz, CDCl_3_) *δ* = −61.06 (s, 3F). HRMS (ESI) calculated for C_22_H_34_F_3_O 371.2556, found 371.2559 [M+H]^+^.

### 3.3. General Conditions for the Des-trifluoromethylation Reaction

The desired PK adduct was dissolved in nitromethane (3 mL/0.1 mmol) in a round bottom flask equipped with a magnetic stirrer and a condenser and the system was put under nitrogen. Water (5 eq.) and DBU (1 eq.) were then added, and the system was heated to reflux until no starting material could be detected by TLC analysis. Upon completion of the reaction, the solvent was removed under vacuum and the crude was purified by silica gel chromatography with mixtures of hexanes/EtOAc of increasing polarity.

(3aS*,4R*,7S*,7aR*)-3-(4-Tolyl)-3a,4,5,6,7,7a-hexahydro-1H-4,7-methanoinden-1-one (**6a**): The general procedure was followed starting from adduct **3a** (77 mg, 0.24 mmol). The reaction proceeded during 12 h and, after purification, the desired product was obtained as a colorless oil (16 mg, 28%). IR (film) *ν*_max._ = 2957, 1688, 1591, 1193 cm^−1^. ^1^H-NMR (400 MHz, CDCl_3_) *δ* = 0.97–1.02 (m, 1H), 1.17–1.11 (m, 1H), 1.50–1.33 (m, 2H), 1.76–1.63 (m, 2H), 2.32–2.29 (m, 1H), 2.39 (d*, J* = 5.5 Hz, 1H), 2.41 (s, 3H), 2.50–2.48 (m, 1H), 3.17–3.14 (m, 1H), 6.59 (d, *J* = 1.1 Hz, 1H), 7.28–7.24 (m, 2H), 7.60–7.56 (m, 2H). ^13^C-NMR (100 MHz, CDCl_3_) *δ* = 21.5 (CH_3_), 28.6 (CH_2_), 29.2 (CH_2_), 31.8 (CH_2_), 38.7 (CH), 39.0 (CH), 50.2 (CH), 55.2 (CH), 127.6 (CH), 129.3 (CH), 129.6 (CH), 130.7 (C), 141.6 (C), 175.0 (C), 210.7 (C) . HRMS (ESI) calculated for C_17_H_19_O 239.1430, found 239.1430 [M+H]^+^.

(3aS*,4R*,7S*,7aR*)-3-(4-Methoxyphenyl)-3a,4,5,6,7,7a-hexahydro-1H-4,7-methanoinden-1-one (**6b**): The general procedure was followed starting from adduct **3b** (55 mg, 0.17 mmol). The reaction proceeded during 12 h and, after purification, the desired product was obtained as a colorless oil (21 mg, 49%). IR (film) *ν*_max._ = 2957, 1678, 1554, 1178 cm^−1^. ^1^H-NMR (400 MHz, CDCl_3_) *δ* = 0.96–1.05 (m, 1H), 1.19–1.10 (m, 1H), 1.51–1.31 (m, 2H), 1.74–1.61 (m, 2H), 2.31 (d, *J* = 4.0 Hz, 1H), 2.38 (dt, *J* = 5.5 and 1.2 Hz, 1H), 2.49 (d, *J* = 3.8 Hz, 1H), 3.13 (d, *J* = 5.3 Hz, 1H), 3.87 (s, 3H), 6.53 (d, *J* = 1.0 Hz, 1H), 6.94 (m, 2H), 7.67–7.62 (m, 2H) . ^13^C-NMR (100 MHz, CDCl_3_) *δ* = 28.6 (CH_2_), 29.2 (CH_2_), 31.8 (CH_2_), 38.6 (CH), 39.1 (CH), 50.2 (CH), 55.2 (CH), 55.4 (CH), 114.3 (CH), 126.0 (C), 128.1 (CH), 129.4 (CH), 161.9 (C), 174.6 (C), 210.5 (C). HRMS (ESI) calculated for C_17_H_19_O_2_ 255.1380, found 255.1378 [M+H]^+^.

(3aS*,4R*,7S*,7aR*)-3-(4-Chlorophenyl)-3a,4,5,6,7,7a-hexahydro-1H-4,7-methanoinden-1-one (**6c**): The general procedure was followed starting from adduct **3c** (19 mg, 0.06 mmol). The reaction proceeded during 12h and, after purification, the desired product was obtained as a colorless oil (4 mg, 30%). IR (film) *ν*_max._ = 2954, 1677, 1593, 1092 cm^−1^. ^1^H-NMR (400 MHz, CDCl_3_) *δ* = 0.99–1.06 (m, 1H), 1.16–1.09 (m,1H), 1.50–1.33 (m, 2H), 1.77–1.60 (m, 2H), 2.30–2.25 (m, 1H), 2.40 (dt, *J* = 5.5 and 1.2 Hz, 1H), 2.50 (d, *J* = 4.1 Hz, 1H), 3.14 (d, *J* = 5.4 Hz, 1H), 6.60 (d, *J* = 1.1 Hz, 1H), 7.45–7.41 (m, 2H), 7.62–7.59 (m, 2H). ^13^C-NMR (100 MHz, CDCl_3_) *δ* = 28.6 (CH_2_), 29.2 (CH_2_), 31.8 (CH_2_), 38.8 (CH), 50.2 (CH), 55.2 (CH), 128.8 (CH), 129.2 (CH), 130.5 (CH), 131.9 (C) 137.1 (C), 173.4 (C), 210.3 (C). HRMS (ESI) calculated for C_16_H_16_ClO 259.0884, found 259.0884 [M+H]^+^.

### 3.4. General Conditions for the Pauson-Khand Reaction with Ethylene

The cobalt complex of the desired alkyne (**2**)was dissolved in anhydrous toluene (7 mL/0.1 mmol complex) in a pressure flask provided with a manometer and a magnetic stirrer. The system was then loaded with ethylene (6 bars) and heated up at 80–85 °C until no starting cobalt complex could be detected by TLC. After that time, the solvent was removed under reduced pressure. The crude was purified by silica gel chromatography using mixtures of hexanes/EtOAc of increasing polarity.

*3-(p-Tolyl)-2-(trifluoromethyl)cyclopent-2-en-1-one* (**5a**): The general procedure was followed starting from cobalt complex **2a** (63 mg, 0.13 mmol). The desired product was isolated as a white solid (17 mg, 53%). Mp: 83–85 °C. IR (KBr) *ν*_max._ = 2978, 1681, 1594, 1092 cm^−1^. ^1^H-NMR (400 MHz, CDCl_3_) *δ* = 2.38 (s, 3H), 2.70 (m, 2H), 2.87 (m, 2H), 7.16 (d, *J* = 8.0 Hz, 2H), 7.22 (d, *J= 8.0 Hz*, 2H). ^13^C-NMR (100 MHz, CDCl_3_) *δ* = 21.3 (CH_3_), 24.08 (CH_2_), 34.0 (CH_2_), 122.5 (q, *^1^J_CF_* = 273 Hz, C), 125.9 (C), 128.8 (q, *^5^J_CF_* = 2 Hz, CH), 128.9 (CH), 139.2 (C), 145.5 (C), 153.4 (C), 206.1 (C). ^19^F-NMR (376 MHz, CDCl_3_) *δ* = −62.82 (s, 3F) .HRMS (ESI) calculated for C_13_H_12_F_3_O 241.0835, found 241.0836 [M+H]^+^.

*3-(4-Methoxyphenyl)-2-(trifluoromethyl)cyclopent-2-en-1-one* (**5b**): The general procedure was followed starting from cobalt complex **2b** (35 mg, 0.07 mmol). The desired product was isolated as a colorless oil (11 mg, 58%). IR (film) *ν*_max._ = 2934, 1725, 1608, 1513, 1259, 1173 cm^−1^. ^1^H-NMR (400 MHz, CDCl_3_) *δ* = 2.69 (m, 2H), 2.87 (m, 2H), 3.83 (s, 3H), 6.94 (m, 2H), 7.25 (m, 2H). ^13^C-NMR (100 MHz, CDCl_3_) *δ* = 24.7 (q, *^4^J_CF_* = 2 Hz, CH_2_), 30.4 (CH_2_), 55.2 (CH_3_), 113.7 (CH), 121.0 (C), 122.6 (q, *^1^J_CF_* = 273 Hz, C), 130.4 (q, *^5^J_CF_* = 2 Hz, CH), 145.0 (q, *^3^J_CF_* = 3 Hz, C), 152.6 (q, *^2^J_CF_* = 34 Hz, C), 160.3 (C), 206.3 (C) . ^19^F-NMR (376 MHz, CDCl_3_) *δ* = −62.76 (s, 3F) . HRMS (ESI) calculated for C_13_H_15_F_3_NO 274.1049, found 274.1045 [M+NH_4_]^+^.

*3-(4-Chlorophenyl)-2-(trifluoromethyl)cyclopent-2-en-1-one* (**6c**)*:* The general procedure was followed starting from cobalt complex **2c** (34 mg, 0.07 mmol). The desired product was isolated as a yellow oil (10 mg, 56%). IR (film) *ν*_max._ = 2917, 1723, 1489, 1257, 1126 cm^−1^. ^1^H-NMR (400 MHz, CDCl_3_) *δ* = 2.71 (m, 2H), 2.90 (m, 2H), 7.21 (m, 2H), 7.39 (m, 2H). ^13^C-NMR (100 MHz, CDCl_3_) *δ* = 24.9 (CH_2_), 34.0 (CH_2_), 127.2 (C), 128.5 (CH), 130.3 (CH), 135.5 (C), 205.4 (C). ^19^F-NMR (376 MHz, CDCl_3_) *δ* = −62.87 (s, 3F). HRMS (ESI) calculated for C_12_H_9_ClF_3_O 261.0289, found 261.0285 [M+H]^+^.

*3-Phenethyl-2-(trifluoromethyl)cyclopent-2-en-1-one* (**6e**)*:* The general procedure was followed starting from cobalt complex **2e** (74 mg, 0.15 mmol). The desired product was isolated as a colorless oil (9 mg, 23%). IR (film) *ν*_max._ = 2933, 1721, 1236, 1129 cm^−1^. ^1^H-NMR (400 MHz, C_6_D_6_) *δ* = 1.45 (m, 2H), 1.59 (m, 2H), 2.35–2.48 (m, 4H), 6.73–6.92 (m, 5H). ^13^C-NMR (100 MHz, C_6_D_6_) *δ* = 24.0 (CH_2_), 25.8 (CH_2_), 32.8 (CH_2_), 34.3 (q, *^4^J_CF_* = 1 Hz, CH_2_), 123.2 (q, *^1^J_CF_* = 273 Hz*,* C), 124.7 (C), 125.9 (C), 126.2 (C), 128.2 (CH), 128.3 (CH), 128.4 (CH), 205.4 (q, *^3^J_CF_* = 20 Hz, C). ^19^F-NMR (376 MHz, C_6_D_6_) *δ* = −64.40 (s, 3F). HRMS (ESI) calculated for C_14_H_14_F_3_O 285.1461, found 285.1461 [M+H]^+^.

## 4. Conclusions

In summary, we have expanded the PKR of dissymmetric trifluoromethyl alkynes to alkenes other than norbornadiene such as norbornene and ethylene. The regioselectivity of these reactions was complete in the case of ethylene, but mixtures of α- and β-trifluoromethyl cylopentenones were detected in the PK adducts of norbornene. The structure of the β-trifluoromethyl cyclopentenones was confirmed by chemical correlation and by NMR analysis.
